# *TCF21* genetic polymorphisms and breast cancer risk in Chinese women

**DOI:** 10.18632/oncotarget.9825

**Published:** 2016-06-05

**Authors:** Xueren Gao, Jiaojiao Yang, Mingxi Wang, Jianqiong Zhang

**Affiliations:** ^1^ Key Laboratory of Developmental Genes and Human Disease, Ministry of Education, Department of Microbiology and Immunology, Medical School of Southeast University, Nanjing, Jiangsu, China; ^2^ Jiangsu Key Laboratory of Molecule Imaging and Functional Imaging, Medical School of Southeast University, Nanjing, Jiangsu, China; ^3^ Department of Medical Oncology, The First Affiliated Hospital of Bengbu Medical College, Bengbu, Anhui, China

**Keywords:** polymorphism, breast cancer, risk

## Abstract

Transcription factor 21 (TCF21) functions as a tumor suppressor and is inactivated in several types of cancer. The purpose of this study is to investigate whether *TCF21* genetic polymorphisms(rs2327429 T>C, rs2327430 T>C, rs2327433 A>G, rs12190287 C>G, rs7766238 G>A, rs4896011 T>A) are associated with the risk of breast cancer in Chinese women. Logistic regression analyses showed that *TCF21* rs12190287 polymorphism was significantly associated with the reduced risk of breast cancer. Stratified analyses based on pathological type indicated that *TCF21* rs12190287 polymorphism was only associated with the reduced risk of infiltrative ductal carcinoma. Real-time quantitative PCR analyses revealed that compared with those carrying rs12190287 CC genotype, subjects with GG genotype had higher expression levels of TCF21 mRNA in normal breast tissues. Furthermore, luciferase activity assay showed that the rs12190287 G allele weakened the binding affinity of hsa-miR-224 to *TCF21* 3′ UTR. These findings suggest that *TCF21* rs12190287 polymorphism can regulate *TCF21* expression and may serve as a potential marker for genetic susceptibility to breast cancer.

## INTRODUCTION

Breast cancer is one of the most frequently diagnosed cancers and the principal cause of cancer deaths among women. It is estimated that more than one million women are diagnosed with breast cancer every year, and more than 410,000 will die of the disease [1]. China accounted for 12.2% of all newly diagnosed breast cancers and 9.6% of all breast cancer-related deaths worldwide [2]. Furthermore, the incidence of breast cancer in China has been steadily increasing for decades, and is expected to increase to more than 100 cases per 10^5^ women aged 55-69 years by 2021 [2–3]. Over the past few decades, a large number of studies have found that environmental factors and genetic background, such as menstrual and reproductive factors, physical activity, alcohol intake and genetic variations, play an important role in the occurrence of breast cancer, but the etiology of breast cancer is extremely complex and has not been fully elucidated [4, 5]. Thus, more studies are needed to identify novel risk factors for breast cancer, which will contribute to risk prediction and prevention of the disease.

Transcription factor 21 (TCF21) is a member of the basic helix-loop-helix (bHLH) transcription factor family that controls development and differentiation of a variety of cell types [6, 7]. Recently, TCF21 has been identified as a tumor suppressor and inactivated in several types of cancer [8–11]. For instance, TCF21 was reported to be frequently silenced in colorectal cancer (CRC) cells and tissues. Restoration of TCF21 expression inhibited CRC cell proliferation, promoted apoptosis and suppressed cell invasion and migration [8]. In addition, TCF21 expression was downregulated in most breast cancer tissues, and TCF21 overexpression could inhibit the proliferation of human breast cancer cell line MDA-MB-231 [11]. It is well-known that single nucleotide polymorphisms (SNPs) in cancer-related genes may affect gene expression through different mechanisms depending on their locations. For instance, a functional SNP (rs10719 T > C) in the 3′ untranslated region (UTR) of DROSHA gene was associated with the risk of bladder cancer by disturbing the binding of hsa-miR-27b and in turn affecting DROSHA expression [12]. There was also one report that an SNP (rs2295080 G > T) in the promoter region of MTOR gene could affect individual susceptibility to renal cell cancer by modulating the endogenous MTOR expression level [13]. Therefore, we hypothesized that SNPs in *TCF21* gene might be associated with breast cancer risk by altering TCF21 expression. To test this hypothesis, we conducted a hospital-based case-control study to evaluate the influence of *TCF21* SNPs on the risk of breast cancer in Chinese women. Subsequently, functional assays were performed to explore the mechanism of the polymorphism conferring individual susceptibility to breast cancer.

## RESULTS

### Associations between *TCF21* polymorphisms and breast cancer risk

The genotype distributions of *TCF21* polymorphisms in cases and controls are shown in Table 1. The observed genotype frequencies of the six polymorphisms in controls conformed to the Hardy-Weinberg equilibrium (HWE) (*P*_HWE_ > 0.05). In the discovery set with 406 cases and 592 controls, genotyping results showed that *TCF21* rs2327429 and rs12190287 polymorphisms were significantly associated with the reduced risk of breast cancer (For rs2327429: C *vs*. T, OR = 0.83, 95%CI = 0.69-0.99, *P* = 0.04; CC *vs*. TT, OR = 0.68, 95%CI = 0.47-0.99, *P* = 0.04. For rs12190287: G *vs*. C, OR = 0.78, 95%CI = 0.65-0.94, *P* = 0.01; GG *vs*. CC, OR = 0.61, 95%CI = 0.41-0.90, *P* = 0.01; GG + CG *vs*. CC, OR = 0.75, 95%CI = 0.58-0.97, *P* = 0.03; GG *vs*. CG + CC, OR = 0.69, 95%CI = 0.48-0.98, *P* = 0.04). To confirm the above findings, associations of *TCF21* rs2327429 and rs12190287 polymorphisms with breast cancer risk were further assessed in the validation set containing 495 cases and 633 controls. The results showed that only *TCF21* rs12190287 polymorphism was associated with the reduced risk of breast cancer (G *vs*. C, OR = 0.82, 95%CI = 0.69-0.97, *P* = 0.02; GG *vs*. CC, OR = 0.68, 95%CI = 0.47-0.97, *P* = 0.03). Furthermore, similar results were also found in pooled analysis (G *vs*. C, OR = 0.80, 95%CI = 0.71-0.91, *P* = 0.001; GG *vs*. CC, OR = 0.64, 95%CI = 0.49-0.84, *P* = 0.001; CG *vs*. CC, OR = 0.81, 95%CI = 0.68-0.98, *P* = 0.03; GG + CG *vs*. CC, OR = 0.77, 95%CI = 0.64-0.92, *P* = 0.003; GG *vs*. CG + CC, OR = 0.72, 95%CI = 0.56-0.92, *P* = 0.007).

**Table 1:** Association between *TCF21* polymorphisms and breast cancer risk

	Cases, *n* (%)	Controls, *n* (%)	Comparison	^a^OR (95% CI)	^a^*P*
Discovery Set	*n* = 406	*n* = 592			
rs2327429			C *vs*. T	**0.83 (0.69-0.99)**	**0.04**
TT	126 (31.0)	154 (26.0)	CC *vs*. TT	**0.68 (0.47-0.99)**	**0.04**
CT	204 (50.2)	302 (51.0)	CT *vs*. TT	0.83 (0.61-1.11)	0.20
CC	76 (18.7)	136 (23.0)	CC + CT *vs*. TT	0.78 (0.59-1.03)	0.08
*P*_HWE_		0.61	CC *vs*. CT + TT	0.77 (0.56-1.06)	0.11
rs2327430			C *vs*. T	0.94 (0.68-1.30)	0.72
TT	341 (84.0)	494 (83.4)	CC *vs*. TT	0.58 (0.11-3.00)	0.51
CT	63 (15.5)	93 (15.7)	CT *vs*. TT	0.98 (0.69-1.39)	0.92
CC	2 (0.5)	5 (0.8)	CC + CT *vs*. TT	0.96 (0.68-1.35)	0.82
*P*_HWE_		0.79	CC *vs*. CT + TT	0.58 (0.11-3.01)	0.51
rs2327433			G *vs*. A	0.95 (0.74-1.21)	0.66
AA	287 (70.7)	415 (70.1)	GG *vs*. AA	0.68 (0.29-1.60)	0.37
AG	111 (27.3)	160 (27.0)	GA *vs*. AA	0.99 (0.75-1.32)	0.97
GG	8 (2.0)	17 (2.9)	GG + GA *vs*. AA	0.97 (0.74-1.28)	0.84
*P*_HWE_		0.74	GG *vs*. GA + AA	0.68 (0.29-1.59)	0.37
rs12190287			G *vs*. C	**0.78 (0.65-0.94)**	**0.01**
CC	163 (40.1)	198 (33.4)	GG *vs*. CC	**0.61 (0.41-0.90)**	**0.01**
CG	190 (46.8)	288 (48.6)	CG *vs*. CC	0.80 (0.61-1.06)	0.12
GG	53 (13.1)	106 (17.9)	GG + CG *vs*. CC	**0.75 (0.58-0.97)**	**0.03**
*P*_HWE_		0.94	GG *vs*. CG + CC	**0.69 (0.48-0.98)**	**0.04**
rs7766238			A *vs*. G	0.91 (0.67-1.23)	0.53
GG	336 (82.8)	484 (81.8)	AA *vs*. GG	0.58 (0.18-1.85)	0.35
AG	66 (16.3)	98 (16.6)	AG *vs*. GG	0.97 (0.69-1.37)	0.86
AA	4 (1.0)	10 (1.7)	AA + AG *vs*. GG	0.93 (0.67-1.30)	0.68
*P*_HWE_		0.06	AA *vs*. AG + GG	0.58 (0.18-1.86)	0.35
rs4896011			A *vs*. T	0.84 (0.61-1.17)	0.31
TT	347 (85.5)	496 (83.8)	AA *vs*. TT	0.36 (0.08-1.69)	0.18
AT	57 (14.0)	88 (14.9)	AT *vs*. TT	0.93 (0.65-1.33)	0.68
AA	2 (0.5)	8 (1.4)	AA + AT *vs*. TT	0.88 (0.62-1.25)	0.47
*P*_HWE_		0.08	AA *vs*. AT + TT	0.36 (0.08-1.70)	0.18
Validation Set	n=495	n=633			
rs2327429			C *vs*. T	0.94 (0.80-1.11)	0.47
TT	155 (31.3)	184 (29.1)	CC *vs*. TT	0.87 (0.62-1.22)	0.43
CT	237 (47.9)	312 (49.3)	CT *vs*. TT	0.89 (0.67-1.17)	0.39
CC	103 (20.8)	137 (21.6)	CC + CT *vs*. TT	0.88 (0.68-1.14)	0.34
*P*_HWE_		0.83	CC *vs*. CT + TT	0.94 (0.71-1.26)	0.69
rs12190287			G *vs*. C	**0.82 (0.69-0.97)**	**0.02**
CC	208 (42.0)	230 (36.3)	GG *vs*. CC	**0.68 (0.47-0.97)**	**0.03**
CG	221 (44.6)	295 (46.6)	CG *vs*. CC	0.82 (0.64-1.06)	0.13
GG	66 (13.3)	108 (17.1)	GG + CG *vs*. CC	0.78 (0.62-1.00)	0.05
*P*_HWE_		0.42	GG *vs*. CG + CC	0.75 (0.54-1.05)	0.09
**Pooled Analysis**	***n* = 901**	***n* = 1225**			
rs2327429			C vs. T	0.89 (0.78-1.00)	0.05
TT	281 (31.2)	338 (27.6)	CC vs. TT	0.79 (0.62-1.01)	0.06
CT	441 (48.9)	614 (50.1)	CT vs. TT	0.86 (0.71-1.06)	0.16
CC	179 (19.9)	273 (22.3)	CC + CT vs. TT	0.84 (0.70-1.02)	0.07
*P*_HWE_		0.85	CC vs. CT + TT	0.87 (0.70-1.07)	0.18
rs12190287			G vs. C	**0.80 (0.71-0.91)**	**0.001**
CC	371 (41.2)	428 (34.9)	GG vs. CC	**0.64 (0.49-0.84)**	**0.001**
CG	411 (45.6)	583 (47.6)	CG vs. CC	**0.81 (0.68-0.98)**	**0.03**
GG	119 (13.2)	214 (17.5)	GG + CG vs. CC	**0.77 (0.64-0.92)**	**0.003**
*P*_HWE_		0.52	GG vs. CG + CC	**0.72 (0.56-0.92)**	**0.007**

a Adjusted for age.

### Stratification analysis of *TCF21* rs12190287 polymorphism with breast cancer risk

The associations of *TCF21* rs12190287 polymorphism with breast cancer risk were further examined with stratification by age, pathological type and tumor stage. As shown in Table 2, stratified analyses based on pathological type indicated that *TCF21* rs12190287 polymorphism was only associated with the reduced risk of infiltrative ductal carcinoma (G *vs*. C, OR = 0.78, 95%CI = 0.68-0.89, *P* < 0.001; GG *vs*. CC, OR = 0.59, 95%CI = 0.45-0.79, *P* < 0.001; CG *vs*. CC, OR = 0.82, 95%CI = 0.67-0.99, *P* = 0.04; GG + CG *vs*. CC, OR = 0.76, 95%CI = 0.83-0.91, *P* = 0.003; GG *vs*. CG + CC, OR = 0.67, 95%CI = 0.51-0.86, *P* = 0.002).

**Table 2:** Stratification analysis of *TCF21* rs12190287 polymorphism with breast cancer risk

Characteristics	G *vs*. C		GG *vs*. CC		CG *vs*. CC		GG + CG *vs*. CC		GG *vs*. CG + CC	
	^a^OR (95% CI)	^a^*P*	^a^OR (95% CI)	^a^*P*	^a^OR (95% CI)	^a^*P*	^a^OR (95% CI)	^a^*P*	^a^OR (95% CI)	^a^*P*
Age (years)										
<50	**0.85 (0.73-0.99)**	**0.04**	0.74 (0.52-1.03)	0.07	0.81 (0.64-1.03)	0.08	**0.80 (0.64-0.99)**	**0.04**	0.83 (0.61-1.13)	0.23
≥50	**0.72 (0.58-0.89)**	**0.002**	**0.52 (0.34-0.80)**	**0.003**	0.82 (0.60-1.13)	0.22	**0.72 (0.54-0.97)**	**0.03**	**0.58 (0.39-0.86)**	**0.01**
Pathological type										
Infiltrative ductal carcinoma	**0.78 (0.68-0.89)**	**<0.001**	**0.59 (0.45-0.79)**	**<0.001**	**0.82 (0.67-0.99)**	**0.04**	**0.76 (0.83-0.91)**	**0.003**	**0.67 (0.51-0.86)**	**0.002**
Other carcinoma	0.93 (0.72-1.21)	0.61	0.91 (0.54-1.52)	0.72	0.80 (0.53-1.20)	0.28	0.83 (0.57-1.21)	0.33	1.03 (0.64-1.64)	0.92
Stage										
I+II	**0.80 (0.70-0.92)**	**0.002**	**0.59 (0.44-0.80)**	**0.001**	0.86 (0.70-1.06)	0.17	**0.79 (0.65-0.96)**	**0.02**	**0.64 (0.49-0.85)**	**0.002**
III+IV	**0.79 (0.65-0.97)**	**0.03**	**0.66 (0.44-0.99)**	**0.05**	**0.57 (0.41-0.79)**	**0.001**	**0.60 (0.44-0.80)**	**0.001**	0.88 (0.60-1.29)	0.51

a Adjusted for age.

### Effects of rs12190287 genotypes on TCF21 mRNA expression levels

**Figure 1:**
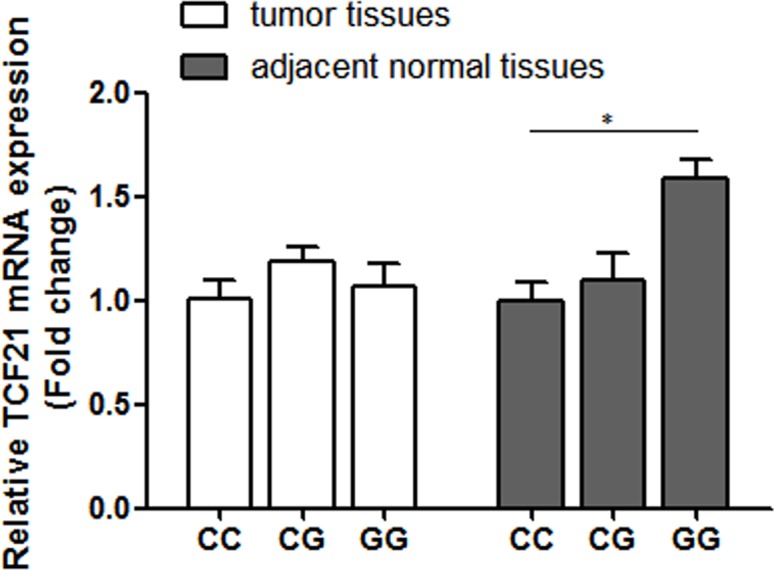
Relative TCF21 mRNA expression in three genotypic groups of rs12190287 (CC, *n* = 16; CG, *n* = 18; GG, *n* = 5) Data represented as mean ± SEM; *P < 0.05.

To assess the effects of rs12190287 genotypes on TCF21 mRNA expression levels, TCF21 mRNA expression levels were detected in 39 tumor tissues and adjacent normal tissues with different genotypes. As shown in Figure 1, TCF21 mRNA levels were significantly higher in normal breast tissues with rs12190287 GG genotypes than in those with rs12190287 CC genotype (*P* < 0.05).

### Bioinformatics analysis of *TCF21* rs12190287 polymorphism

Bioinformatics tools, including TargetScan and miRanda, were used to predict potential function of rs12190287 polymorphism in the 3′UTR region of the *TCF21* gene. As shown in Figure S1, rs12190287 polymorphism was located within the seed region (2 to 8 bp from 5′ end of miRs) of *TCF21* 3′ UTR and hsa-miR-224 hybridization, and the presence of the rs12190287 G allele may disrupt the binding of hsa-miR-224 and *TCF21* 3′ UTR.

### Luciferase activity

**Figure 2:**
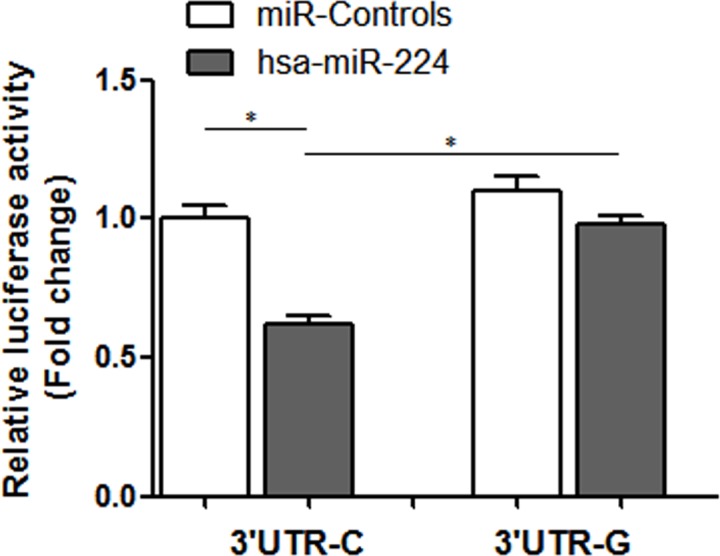
Relative luciferase activity of variant allele on luciferase reporter genes bearing human TCF21 3′ UTR in 293T Data represented as mean ±SEM; *P < 0.01.

As shown in Figure 2, compared with the pmirGLO-TCF21 3′ UTR-G, the relative luciferase activity of pmirGLO-TCF21 3′ UTR-C was significantly reduced in the presence of hsa-miR-224, suggesting that rs12190287 polymorphism can regulate TCF21 mRNA expression by disturbing hsa-miR-224 binding.

## DISCUSSION

MicroRNAs (miRNAs) are a class of evolutionarily conserved, single-stranded, non-coding RNA molecules (18~22 nucleotides), and regulate the expression of roughly 10%-30% of all human genes [14]. Accumulating evidence has linked the dysregulated miRNA expression to cancer [15–17]. For instance, miR-145 was downregulated in non-small cell lung cancer tissues, which was correlated with late clinical stage and poorly differentiated carcinoma [15]. MiR-99b was expressed at high levels in tissues of patients with hepatocellular carcinoma (HCC) and in cell lines derived from HCCs. Elevated levels of miR-99b correlated with poor survival of patients with HCC [16]. In addition, a recent study found that hsa-miR-224 expression was significantly upregulated in breast cancer cell lines, especially in highly invasive MDA-MB-231 cells. Hsa-miR-224 could inhibit the expression of tumor suppressor gene RKIP by directly targeting its 3′ UTR, and contribute to breast cancer cell metastasis [17]. However, there is increasing evidence that genetic variation in miRNA binding site can disrupt miRNA-mediated regulation of gene expression [18, 19]. For instance, FOXO1 rs17592236 C > T polymorphism was associated with reduced risk of HCC. Functional dual luciferase reporter assays verified that the rs17592236 polymorphism was a target site of hsa-miR-137 and modulated the binding affinity of hsa-miR-137 to FOXO1 3′ UTR [18]. SCRN1 rs6976789 C > T polymorphism located in miR-148a target site, and the rs6976789 variant T allele enhanced the binding ability of miR-148a [19]. In the current study, we investigated the associations of the *TCF21* polymorphisms (rs2327429 T > C, rs2327430 T > C, rs2327433 A > G, rs12190287 C > G, rs7766238 G > A, rs4896011 T > A) with the risk of breast cancer in Chinese women, and found that the *TCF21* rs12190287 polymorphism in hsa-miR-224 binding site was significantly associated with the reduced risk of breast cancer under all comparison models. Intriguingly, stratified analyses based on pathological type indicated that *TCF21* rs12190287 polymorphism was only associated with the reduced risk of infiltrative ductal carcinoma under all comparison models. In addition, *in vitro*and*in vivo*studies indicated that rs12190287 polymorphism had an allele-specific effect on TCF21 mRNA expression. The rs12190287 C allele had a lower transcription activity than G allele, which supported the hypothesis that functional genetic variants in 3′ UTR region could influence miRNA-mediated regulation of gene expression.

To the best of our knowledge, this was the first data on the epidemiology providing comprehensive insights into the effects of *TCF21* polymorphisms on the risk of breast cancer. Although some interesting results were found, several limitations should be considered when interpreting our results. For instance, inherent selection bias may exist because the cases and controls were from the hospital. Therefore, the findings of this study should be validated in the future through a population-based study. Furthermore, environmental factors and lifestyle, including ionizing radiation, alcohol consumption and high-fat diets, may interact with *TCF21* polymorphisms and were not included in this analysis [20–22].

Taken together, our findings suggest that TCF21 rs12190287 polymorphism can regulate TCF21 expression and may serve as a potential marker for genetic susceptibility to breast cancer in Chinese women.

## MATERIALS AND METHODS

### Study subjects

A total of 901 patients with histopathologically verified breast cancer and 1225 healthy women were consecutively recruited between October 2013 and December 2015 at the Affiliated Hospital of Bengbu Medical College. The selection criteria for all breast cancer patients were that they had no prior history of any cancers and reported no family history of breast cancer. The selection criteria for healthy women who underwent a hospital-based physical examination, were that they had no history of cancer, and were frequency matched to cases on age. In addition, 39 tumor tissues and adjacent normal tissues from the untreated breast cancer patients were collected from the Affiliated Hospital of Bengbu Medical College between October 2013 and March 2014. All subjects were ethnically homogenous Han Chinese from the same geographical region. Clinical information was acquired from medical records and pathology reports (Table S1). Written informed consent was obtained from each subject. The research protocol was approved by the Ethics Committee of the Affiliated Hospital of Bengbu Medical College.

### SNPs selection

Haploview 4.2 and HapMap database of Han Chinese in Beijing (Release 27) was applied to obtain SNPs with a minor allele frequency of more than 5% in the *TCF21* locus. A total of ten SNPs were obtained in the region of *TCF21* stretching from 2 kb upstream of the transcriptional start site to the transcriptional stop site (Figure S2). After performing linkage disequilibrium analysis by the Haploview program, we found six tag SNPs that captured all the alleles with a mean r^2^ of 0.98 (Table S2). Therefore, these SNPs were selected to investigate associations between *TCF21* polymorphisms and breast cancer risk.

### DNA extraction and genotyping

Genomic DNA was extracted from peripheral blood lymphocytes of all the studied subjects by a standard salting-out method. Genotypes were determined by the polymerase chain reaction-ligase detection reaction (PCR-LDR) method. The sequences of PCR primers and LDR probes are summarized in Table S3 and Table S4, respectively. Three LDR probes, including two allele specific probes and one common fluorescent labeled probe, were synthesized for each SNP locus. The LDR parameters were as follows: 94°C for 2 min, 30 cycles at 94°C for 30 s and 56°C for 3 min. The fluorescent products of LDR were analyzed on ABI 3730xl DNA Analyzer (Applied Biosystems, Foster City, CA, USA). To evaluate the reliability of LDR assays, thirty samples were randomly selected and re-genotyped by DNA sequencing. The results were 100% concordant.

### Real-time quantitative PCR analysis

Total RNA was isolated from tissue samples using TRIzol reagent following RNase-Free DNase treatment (Invitrogen, Carlsbad, CA, USA). cDNA were synthesized from total RNA using the PrimeScript RT reagent kit with gDNA Eraser (Takara, Otsu, Japan) according to the manufacturer's instructions. The mRNA levels were measured by SYBR green real-time quantitative PCR on a StepOnePlus™ Real-time PCR System. β-actin was chosen as the endogenous control to normalize gene expression. Quantitative PCR primer sequences used for TCF21 and β-actin were shown in Table S5. Relative quantification of TCF21 mRNA was calculated by using the 2^−ΔΔCt^ method, and each assay was done in triplicate. In all cases, the relative lower TCF21 expression group was used as calibrator (fold change = 1). A melting curve analysis was performed for the PCR products to evaluate primer specificity.

### Bioinformatics analysis of *TCF21* rs12190287 polymorphism

TargetScan and miRanda were used to predict potential function of rs12190287 polymorphism in the 3′ UTR region of the *TCF21* gene. The human *TCF21* 3′ UTR containing rs12190287 polymorphism was identified according to the UCSC genome browser (http://genome.ucsc.edu). The mature human miRNA sequences were obtained from the miRBase database (http://www.mirbase.org/).

### Dual-luciferase reporter assay

The human *TCF21* 3′ UTR fragments with rs12190287 C allele or G allele were directly synthesized and then respectively inserted into the multiple cloning site of the pmirGLO vector (Promega, Madison, WI, USA) [23]. After cloning, amplification, and DNA sequence confirmation, these vectors were designated as pmirGLO-TCF21 3′ UTR-C and pmirGLO-TCF21 3′ UTR-G, respectively. 293T cells were seeded at 1 × 10^5^ cells per well in 24-well plates. When cells reached 80% confluence, they were transiently co-transfected with recombinant reporter plasmids and chemically synthesized hsa-miR-224 or negative control miRNA (RiboBio, Guangzhou, China) using Lipofectamin 2000 (Invitrogen, Carlsbad, CA, USA) following manufacturer's instructions. Twenty-four hours after transfection, cells were harvested by the addition of 100 μl passive lysis buffer. Luciferase activity was measured by the dual-luciferase reporter assay system (Promega, Madison, WI, USA) and expressed as the ratio of Firefly luciferase to Renilla luciferase activities. In addition, the relative luciferase activity was normalized to 3′ UTR-C + miR-Controls, shown as fold change. Three independent transfection experiments were performed, and each luciferase assay was done in triplicate with the same conditions.

### Statistical analysis

All data were analyzed with SPSS version 19.0 (SPSS Inc., Chicago, IL, USA). Differences in the distributions of age between cases and controls were detected using chi-square (χ^2^) test. A goodness-of-fit χ^2^ test was used to evaluate HWE in controls. Associations of *TCF21* polymorphisms with breast cancer risk were measured by the odds ratio (OR) and its corresponding 95% confidence interval (CI), and estimated using a logistic regression model. All ORs and 95% CIs were adjusted for age when it was appropriate. Student's *t*-test was used to compare the difference in luciferase reporter gene expression. One-way ANOVA test were used to analyze the difference of TCF21 mRNA levels among different genotypes. A *P* value of less than 0.05 was considered statistically significant, and all statistical tests were two-sided.

## SUPPLEMENTARY MATERIALS


