# Supplementary material for: TCF21 genetic polymorphisms and breast cancer risk in Chinese women
Source: Oncotarget. 2016 Jun 5;7(34):55757–64. doi: 10.18632/oncotarget.9825 (PMC5342451; doi:10.18632/oncotarget.9825)
Supplement: Supplementary file 1 [file oncotarget-07-55757-s001.pdf]

TCF21 genetic polymorphisms and breast cancer risk in Chinese women

Supplementary Material

Table S1: Main characteristics of breast cancer cases and cancer-free controls.

Characteristics	Cases, n (%)	Controls, n (%)	*P*
Total	901	1225	
Age (years)			
<50	573 (63.6)	785 (64.1)	0.82
≥50	328 (36.4)	440 (35.9)	
Pathological type			
Infiltrative ductal carcinoma	771 (85.6)		
Other carcinoma	130 (14.4)		
Stage			
Ⅰ	223 (24.8)		
Ⅱ	442 (49.1)		
Ⅲ	177 (19.6)		
Ⅳ	59 (6.5)		

Table S2: Main characteristics of tag SNPs in the *TCF21* gene.

Tag SNPs	Location	Allele	MAF	Captured SNP (r^2)
rs2327429	Promoter	C/T	0.48	-
rs2327430	Promoter	C/T	0.06	-
rs2327433	Intron	A/G	0.14	rs3734281 (1.0)
rs12190287	3' UTR	C/G	0.41	-
rs7766238	3' UTR	G/A	0.10	rs1572425 (0.93), rs2327431 (0.93), rs9389114 (0.93)
rs4896011	3' UTR	T/A	0.08	-

MAF, minor allele frequency; 3' UTR, 3' untranslated region.

Table S3: PCR primers sequences used for amplification of tag SNPs-containing regions.

TagSNPs	Primers sequences (5’-3’)
rs7766238, rs4896011, rs12190287	Forward: TAGGCTGGTCCCGACTGGAGA
	Reverse: ATGGATGGCCTGCCTGGAATC
rs2327433	Forward: CCGCAGTGTTTACAAGACGTCT
	Reverse: CTCTCGCTCCAGGTACCAAAC
rs2327430, rs2327429	Forward: TTCCATCACCATAAAGATTCT
	Reverse: CGGAGGGAAACTCAATGCACA

Table S4: LDR probes sequences used for genotyping.

TagSNPs	Probes	Probes sequences (5’-3’)
rs7766238	Common	CCCTTCCATCACCCCACCACCGCCA- FAM
	A specific	GAGGACTTTTCAAGATGTCAGGATA
	G specific	TTTGAGGACTTTTCAAGATGTCAGGATG
rs2327433	Common	AGCCTTGAAACTTCCGCTTCATTTG- HEX
	A specific	ATTTATACTGTTATTCCTCGCAGAA
	G specific	TTTATTTATACTGTTATTCCTCGCAGAG
rs2327430	Common	CCGGCCACAGTTGGGAGAAGGTGGCTTT- HEX
	C specific	TTTTGGACAGAACATGCTGCTTCTCGGCC
	T specific	TTTTTTTGGACAGAACATGCTGCTTCTCGGCT
rs4896011	Common	TGCCTTTCACGTTTCGCAAATTTTCCAATCTC- FAM
	A specific	TTTTTTTTAGAGTCCCCAGTGTGATTCAATTGA
	T specific	TTTTTTTTTTTAGAGTCCCCAGTGTGATTCAATTGT
rs2327429	Common	TGGCAGGATGCGGTTGATACTCCCTTTTTTT- HEX
	C specific	TTTTTTTTAAAGCCTTGGAGTCTTATGAGGTGC
	T specific	TTTTTTTTTTTAAAGCCTTGGAGTCTTATGAGGTGT
rs12190287	Common	TTCATCCACCTGTCTATTTGCACATTTT- FAM
	C specific	TTTTTCCAAGGGCTGAGAACTTCGGTGAC
	G specific	TTTTTTTTCCAAGGGCTGAGAACTTCGGTGAG

Table S5: Quantitative PCR primers sequences used for TCF21 and β-actin.

Gene	Primers sequences (5’-3’)
TCF21	Forward: GCCTTCTCCAGACTCAAGACCAC
	Reverse: CATAAAGGGCCACGTCAGGTTG
β-actin	Forward: GTCATTCCAAATATGAGATGCGT
	Reverse: GCTATCACCTCCCCTGTGTG

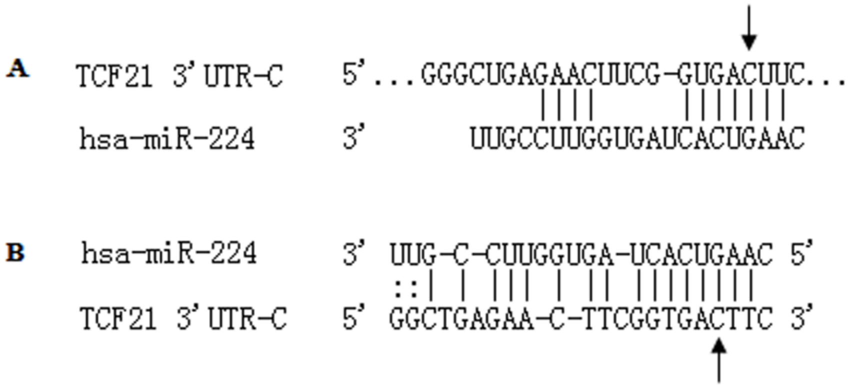

Figure S1: Bioinformatics analysis of *TCF21* rs12190287 polymorphism (A: TargetScan, B: miRanda).

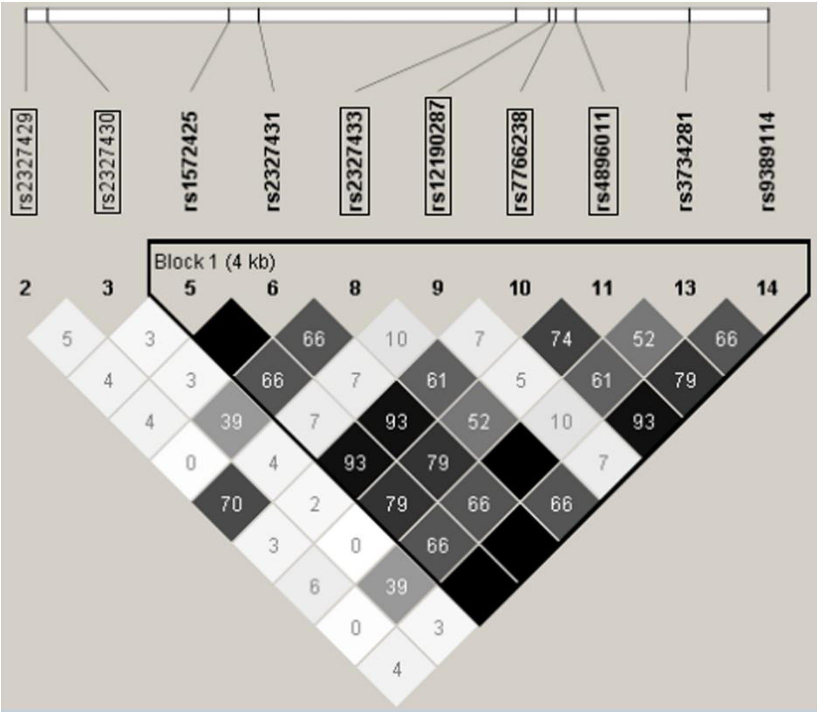

Figure S2: Linkage disequilibrium plot of *TCF21* SNPs with a minor allele frequency of more than 5%.
